# A Single 9-Colour Flow Cytometric Method to Characterise Major Leukocyte Populations in the Rat: Validation in a Model of LPS-Induced Pulmonary Inflammation

**DOI:** 10.1371/journal.pone.0142520

**Published:** 2016-01-14

**Authors:** Ashton Barnett-Vanes, Anna Sharrock, Mark A. Birrell, Sara Rankin

**Affiliations:** 1 Inflammation, Repair and Development Group, National Heart and Lung Institute, Imperial College London, London, United Kingdom; 2 Centre for Blast Injury Studies, Imperial College London, London, United Kingdom; 3 Academic Department of Military Surgery and Trauma, Royal Centre for Defence Medicine, Birmingham, United Kingdom; 4 Respiratory Pharmacology Group, National Heart and Lung Institute, Imperial College London, London, United Kingdom; Virgen Macarena University Hospital, School of Medicine, University of Seville, SPAIN

## Abstract

The rat is a commonly used model for immunological investigation. Yet basic research and characterisation of leukocyte populations and sub-sets lags far behind murine research, with inconsistency on reported leukocyte markers and their overlap. These shortcomings limit the opportunity for more complex and advanced rat immunology research. In this study, we developed a robust 9-colour flow-cytometric protocol to elucidate the major blood and tissue rat leukocyte populations, and validated it in a model of LPS-induced pulmonary inflammation. Blood and tissues (lung, BALF, spleen, liver, bone marrow) from naïve Sprague-Dawley rats were collected and analysed by flow cytometry (FCM). Rats were exposed to aerosolised saline or LPS (1mg/mL), at 3 and 24hrs thereafter blood, lung and BALF were collected and analysed using FCM and ELISA. Neutrophils, two monocyte subsets, NK Cells, B Cells, CD4+, CD8+ T Cells and alveolar macrophages can be identified simultaneously across different tissues using a 9-colour panel. Neutrophils and monocytes can be distinguished based upon differential expression of CD43 and His48. Neutrophils and CD43Lo/His48Hi monocyte-macrophages are elevated in the lung at 3 and 24hrs during LPS-induced pulmonary inflammation. This validated method for leukocyte enumeration will offer a platform for greater consistency in future rat immunology and inflammation research.

## Introduction

Reproducible and robust characterisation of leukocytes is necessary for advanced immunological and inflammation research. Studies conducted in rat are currently limited by the lack of an accurate and validated method that permits simultaneous quantification of major leukocyte subsets.

Neutrophils are the primary first responder to inflammation [1]. Following release from the bone marrow these cells are capable of circulating in the periphery [2], marginating in the vasculature or being recruited into tissues through a complex chemokine and cytokine cascade facilitated by endothelial and epithelial cells [2]. In the rat they are commonly distinguished by expression of the RP-1 antigen [3] or His48 together with high-granularity [4].

Monocytes are another early responder to sites of local and systemic inflammatory responses [5]. Two circulating subsets are observed in humans and rodents [6]. In humans these are characterised by differential CD14 and CD16 expression [7], in mice by CCR2, CX3CR1 and Ly6C [8,9] and in rats by CD43 expression [10]. In rats, CD43 Hi and Lo monocytes are thought to be analogous to the Ly6C Lo (Non-classical) and Hi (Classical) murine monocytes respectively [11,12]. Macrophages play key roles in tissue homeostasis and inflammation. Believed to originate from embryonic progenitors, they possess the ability for self-renewal, thus enabling maintenance of the macrophage pool in adulthood [5]. In rats, CD68 (ED1) is considered to be a ‘pan-macrophage’ marker [13].

Natural Killer (NK) cells are responsible for surveying and responding to stressed or abnormal tissue and are capable of activating or supressing inflammatory responses by interacting with macrophages, T Cells and Dendritic Cells (DCs) [14]. In the rat they are recognised by CD161 (NKR-P1) [4]. T Cells and B Cells play key roles in anti-viral, bacterial and cancer responses. T Cells are commonly distinguished by either their CD3 or T Cell Receptor (TCR) expression, with co-expression of CD4 or CD8 characterising ‘helper’ and ‘cytotoxic’ sub-populations respectively, or Foxp3 and CD25 for ‘regulatory’ T Cells [15]. B Cells are identified by expression of CD45RA [16], a uniquely expressed isoform of the common leukocyte antigen.

However, limitations in the availability of antibodies and choice of different fluorochromes means investigators must either perform costly repeated staining and analysis for different populations or attempt to elucidate multiple populations with few markers; compromising the accuracy of findings. The aim of this study was to develop a simple and reproducible method to simultaneously examine rat leukocyte populations across different tissues by flow cytometry (FCM). We then validated this method in a well-characterised model of Lipopolysaccharide (LPS) induced pulmonary inflammation.

## Methods

### Animals and ethics

Sprague-Dawley female rats (180–200g) were purchased from Charles River (Kent, UK). Food and water were supplied ad libitum. Guidelines for animal welfare were strictly observed under the Scientific Procedures Act 1986 Home Office, United Kingdom. Euthanasia was performed under license either by overdose intraperitoneal injection of pentobarbitone or overdose of CO^2^ inhalation. This study was conducted under Home Office Institutional and departmental License for which full ethical approval is granted.

### LPS aerosolised inhalation

Rats were simultaneously exposed in separate chambers to aerosolised LPS (Sigma-Aldrich, USA) 1mg/ml or sterile saline (Fresenius Kabi, UK) for 30 minutes. At 3 and 24hrs thereafter, rats were euthanised by anaesthetic overdose of intra-peritoneally (i.p) injected Pentobarbitone. Naïve rats used in this study were euthanised by overdose of CO^2^ inhalation.

### Tissue collection

Unless otherwise stated, centrifugations were performed at 4°C and media used throughout was RPMI (Sigma-Aldrich) containing 10% Fetal Bovine Serum (FBS) (Invitrogen, USA). After overdose, blood was collected in citrated tubes on ice from the right femoral vein and centrifuged at 1300g for 10 mins. Plasma was collected and stored at -80°C for further analysis. Lungs were lavaged twice using 3mL of ice-cold RPMI each with a 30 second pause before collection. Broncho-alveolar lavage fluid (BALF) was centrifuged at 800g for 5 mins, supernatant was stored at -80°C and cells were resuspended on ice. For FCM, right inferior lung lobes, superior caudate liver lobes and spleen were collected in media on ice. Bone marrow was flushed from the left tibia using media, cells were centrifuged at 800g and resuspended on ice.

### Tissue processing

Lung and liver lobes were cut by scissor into smaller fragments and suspended in media containing tissue digestion enzymes; DNAse I 5mg/mL, Collagenase D 30mg/mL and Dispase II 5mg/mL (all from Roche, Switzerland). Tissue was incubated in digestion buffer in a water bath with shaking at 37°C for 30 minutes. After, liver cells were resuspended in 36% Percoll (Sigma-Aldrich) (adapted from [17]) and centrifuged for 10mins at 500g without brake to separate leukocytes from fibrous tissue. Spleen was cut into smaller fragments but not subjected to enzymatic digestion. All tissues were strained using a 40μM nylon mesh (Fisher Scientific, USA). Red blood cells were lysed using Ammonium-Chloride-Potassium buffer as previously described [18]. Cell pellets were resuspended and viable cells counted using Trypan blue staining solution on a haemocytometer (Nikon, Tokyo Japan). Cytospins were performed on glass covered slips stained with Wright-Geimsa stain (Sigma-Aldrich) and imaged using a light microscope (Leica, Germany).

### Flow cytometry

Four lasers and 9 different PMT channels were utilised for the 9-colour staining panel; cells were stained with antibodies as detailed in Table 1. Briefly, cells were washed and stained with Live/Dead dye (eBioscience) in PBS. Cells were blocked with anti-CD32 to prevent Fc-mediated non-specific binding as per manufacturer’s instructions. Cells were then stained with antibodies in buffer containing PBS, 1% Bovine Serum Albumin (BSA) and 0.1% Sodium Azide at 4°C for 30 mins followed by further washing and cell fixation (BD Cell Fix). For monocyte surface antigen characterisation, cells were stained with the baseline antibodies detailed in Table 2. Cells were then stained with all antibodies listed in Panel 1 and each sample individually with a Mab listed in Panel 2. Flow cytometric compensation was performed using fluorescent compensation beads (OneComp eBeads, eBioscience USA) and cells were analysed using a multi-parameter flow cytometer (Fortessa LSR BD Biosciences, USA). Cell sorting was performed using a Fortessa Aria (BD Biosciences, USA). For identification of positive and negative populations, the fluorescence minus one (“FMO”) principle was utilised to account for background antibody fluorescence.

**Table 1:** Monoclonal antibodies for general leukocyte FCM.

Antibody	Fluorochrome	Supplier	Clone	Dilution	Laser	Filter
Live-Dead	eFluor® 780	eBioscience		1 in 1000	Red (640nm)	780/60
CD32	N/A	BDPharmingen	D34-485	1 in 200		
CD45	Alexa-Fluor® 700	Biolegend	OX-1	1 in 100	Red (640nm)	730/45
CD3	VioGreen	Miltenyi Biotec	REA223	1 in 100	Violet (405nm)	525/50
CD4	V450	BDBioscience	OX-35	1 in 200	Violet (405nm)	450/50
CD8	PerCP-eFluor® 710	eBioscience	OX8	1 in 200	Blue (488nm)	695/40
CD43	PE	Biolegend	W3/13	1 in 200	Yellow-Green (561nm)	585/15
His48	FITC	eBioscience	HIS48	1 in 200	Blue (488nm)	530/30
CD161	APC	Biolegend	3.2.3	1 in 200	Red (640nm)	670/14
CD45R (B220)	PE-Cy7	eBioscience	HIS24	1 in 200	Yellow-Green (561nm)	780/60

**Table 2:** Monoclonal antibodies for monocyte surface antigen characterisation.

	Antibody	Fluorochrome	Supplier	Clone	Dilution
**Baseline Mabs**					
	Live-Dead	eFluor® 780	eBioscience		1 in 1000
	CD32	N/A	BDPharmingen	D34-485	1 in 200
	CD3	VioGreen	Miltenyi Biotec	REA223	1 in 100
	CD43	PE	Biolegend	W3/13	1 in 200
	His48	FITC	eBioscience	HIS48	1 in 200
**Panel 1**					
	MHCII	PerCP-eFluor® 710	eBioscience	OX17	1 in 200
	CD11b	V450	BDBioscience	WT.5	1 in 200
	CD172a	Alexa-Fluor® 700	Novus Biologicals	OX-41	1 in 50
**Panel 2**					
	CD163	Alexa Fluor® 647	Serotec	ED-2	1 in 50
	CD86	Alexa Fluor® 647	Biolegend	24F	1 in 50
	CD11c	Alexa Fluor® 647	Serotec	8A2	1 in 50

### ELISA

BALF was thawed and analysed using a multiplex ELISA (MesoScaleDiscovery Maryland, USA) according to the manufacturer’s instructions.

### Data & Statistics

All data were collected from 2–3 individual experiments expressed as mean ± standard error of mean (SEM). Flow cytometric data were analysed using FlowJo v7.6.5 (Tree Star Inc, USA). Statistical data analysed using a non-parametric Mann Whitney t-test using GraphPad Prism v5 (San Diego, USA), *p<0.05, **p<0.01.

## Results

### Leukocyte panel and distribution

**Fig 1:**
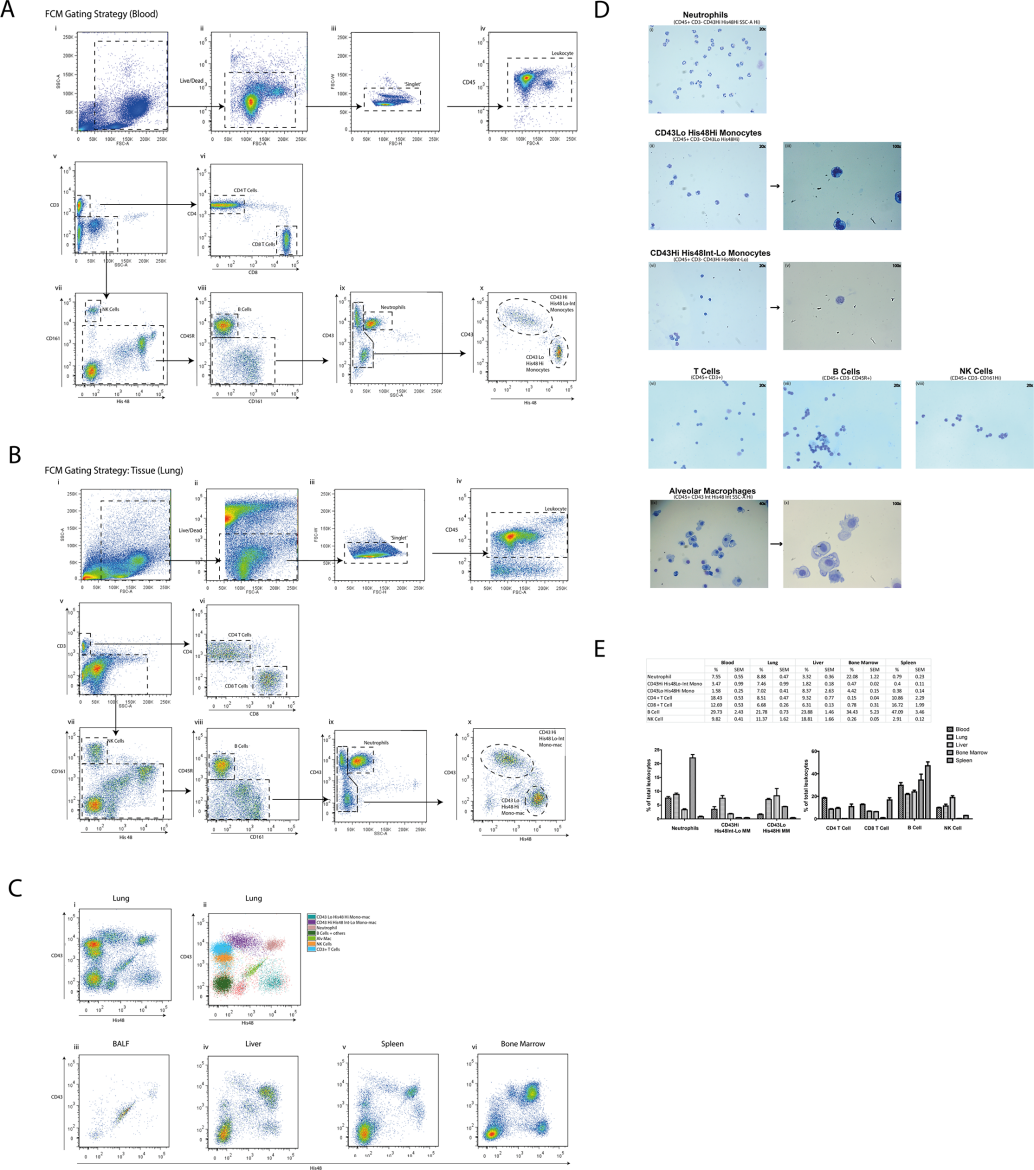
A 9-colour flow cytometric panel of major leukocyte subset discrimination in the rat. Rat blood (A) and lung tissue (B) were collected and processed for FCM staining with antibodies listed in Table 1. Cells from the lung were then back-gated to show cellular profiles according to specific antibody expression presented using CD43 and His48 expression, together with the corresponding distribution profiles for BALF, liver, spleen and bone marrow (C). Leukocytes from the blood and AlvMacs from the lung were then sorted and images of cytospins stained with wright-geimsa were obtained (D). Blood, lung, liver, spleen and bone marrow were processed, analysed and the relative proportion of each leukocyte population presented (E) where n = 3 naïve rats from a single experiment presented mean ± SEM.

Using antibodies targeted against a range of surface antigens (Table 1A) our gating strategy in blood (Fig 1A) first removed debris (i) and cells that were not: ‘live’ (ii), singlets (iii) or CD45+ (iv). To remove singlets, we utilised the FSC-H and FSC-W parameters to identify and exclude cells stuck together (doublets) and larger clumps. This resulted in a population of live single leukocytes which were then sequentially separated, first for T lymphocytes by CD3 (v) which were further delineated by CD4 and CD8 (vi). NK cells were identified by CD161 (vii) and B Cells by CD45R (viii). We took advantage of the differential expression of CD43 and higher granularity of neutrophils (ix) to separate them from two monocyte subsets (CD43Lo/His48Hi and CD43Hi/His48Int-Lo) which demonstrated reciprocal expression for CD43 and His48 (x). The same gating strategy was applied successfully to lung tissue (Fig 1B i-x), we were able to identify Alveolar Macrophages based on their unique pattern of autofluorescence. Fig 1C shows the relative distribution of back-gated cell populations in lung tissue (i,ii) according to CD43 and His48 expression, and their respective distribution in BALF (iii), liver (iv), spleen (v) and bone marrow (vi). To validate the specificity of our panel, we sorted neutrophils, monocytes, lymphocytes and NK cells from the blood and AlvMacs from the lung, before preparation as cytospins for morphological examination by light microscopy using Geimsa staining (Fig 1D). All cells demonstrated the typical morphology expected. We noted that CD43Lo/His48Hi monocytes appeared to show ‘kidney-bean’ shaped nuclei, compared to CD43Hi/His48Int-Lo which exhibited a larger more spherical nuclei. Finally, we compared the relative proportion of cells recognised in our FCM panel in naïve rats (Fig 1E) as a percentage of CD45+ cells. We did not examine cell populations in the BAL due to the over-abundance of alveolar macrophages in naïve rats.

### Monocyte characterisation

**Fig 2:**
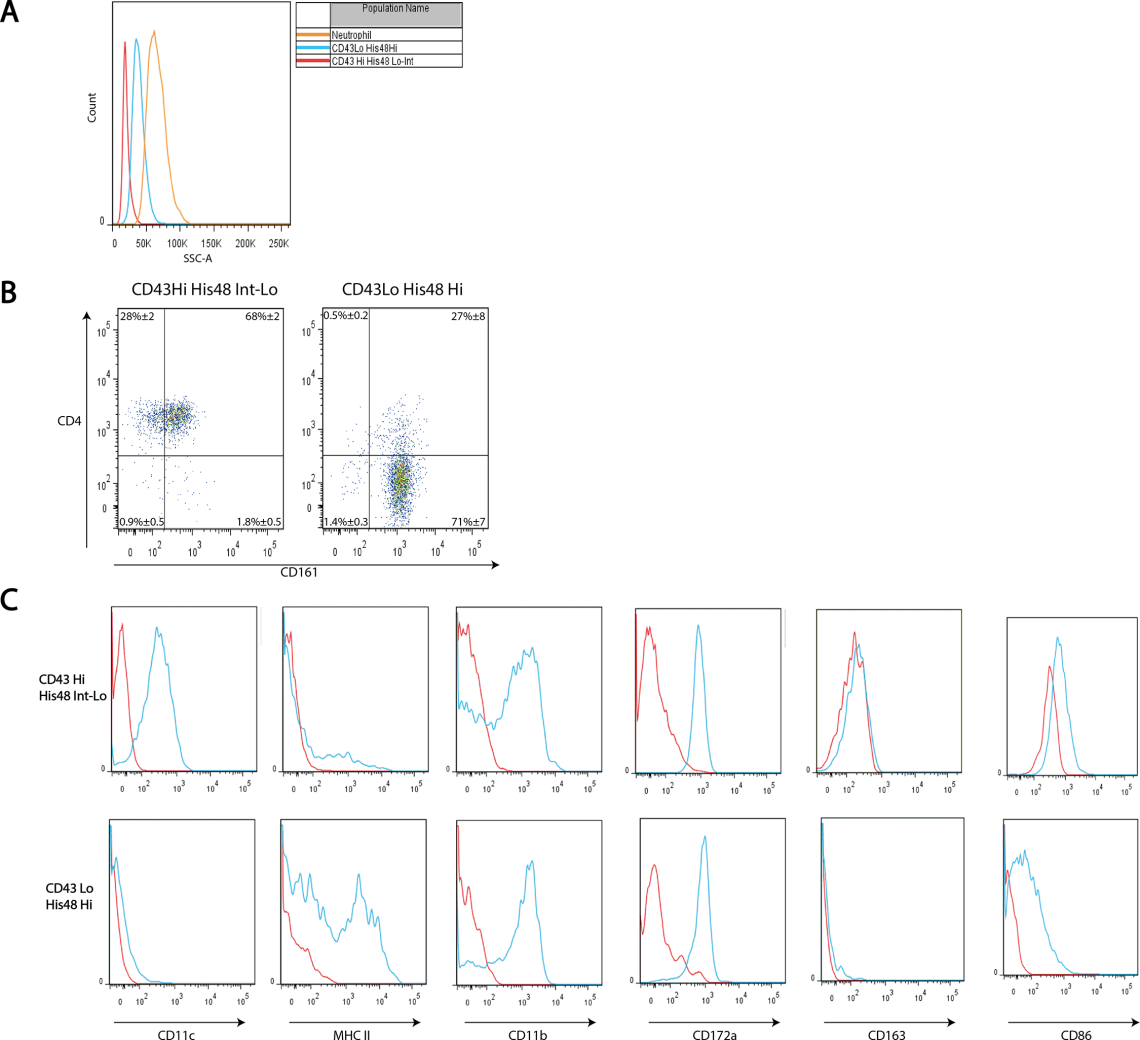
Rat monocyte subsets differ in granularity and cell-surface antigen expression. ‘CD43lo-His48Hi’ and ‘CD43Hi-His48Int-Lo’ blood monocytes and neutrophils were analysed by FCM for granularity by mean side-scatter (A). CD4 and CD161 expression was examined by FCM, with gates drawn according to FMO’s, quadrants presented as mean ± SEM from 3 rats (B). Surface expression of CD11c, MHC II, CD11b, CD172a, CD163 and CD86 were determined in relation to FMO’s by FCM, histograms are representative. Red histograms show the gated population’s FMO, blue histograms show the gated population’s fluorescence (C).

Monocyte populations were further characterised using antibodies detailed in Table 2. We show (Fig 1A) that His48 is an additional marker that exhibits reciprocal expression to CD43 on monocytes. Fig 2A shows an increased granularity (SSC-A) of CD43Lo/His48 Hi monocytes compared to CD43Hi/His48Int-Lo, but these monocytes are still less granular than neutrophils. CD43Hi/His48Int-Lo monocytes are uniformly CD4 positive, with 68%±2 of cells positive for CD161. Conversely, 71%±7 of CD43Lo/His48Hi monocytes were CD4 negative, 98% were CD161 positive (Fig 2B). We then examined the expression of each monocyte subset for CD11c, MHCII, CD11b, CD172a, CD163, and CD86 (Fig 2C). We found CD43Hi/His48Int-Lo monocytes were CD11c positive and MHC II negative, while CD43Lo/His48Hi monocytes were CD11c negative and MHC II positive. Both monocyte subsets were uniformly positive for CD11b, CD172a and CD86 and negative for CD163. Taken together our data show that a FCM strategy using CD43 in combination with His48 can effectively distinguish two-distinct populations of monocytes, and be utilised to discriminate major leukocyte populations across blood and tissue.

### LPS Model

**Fig 3:**
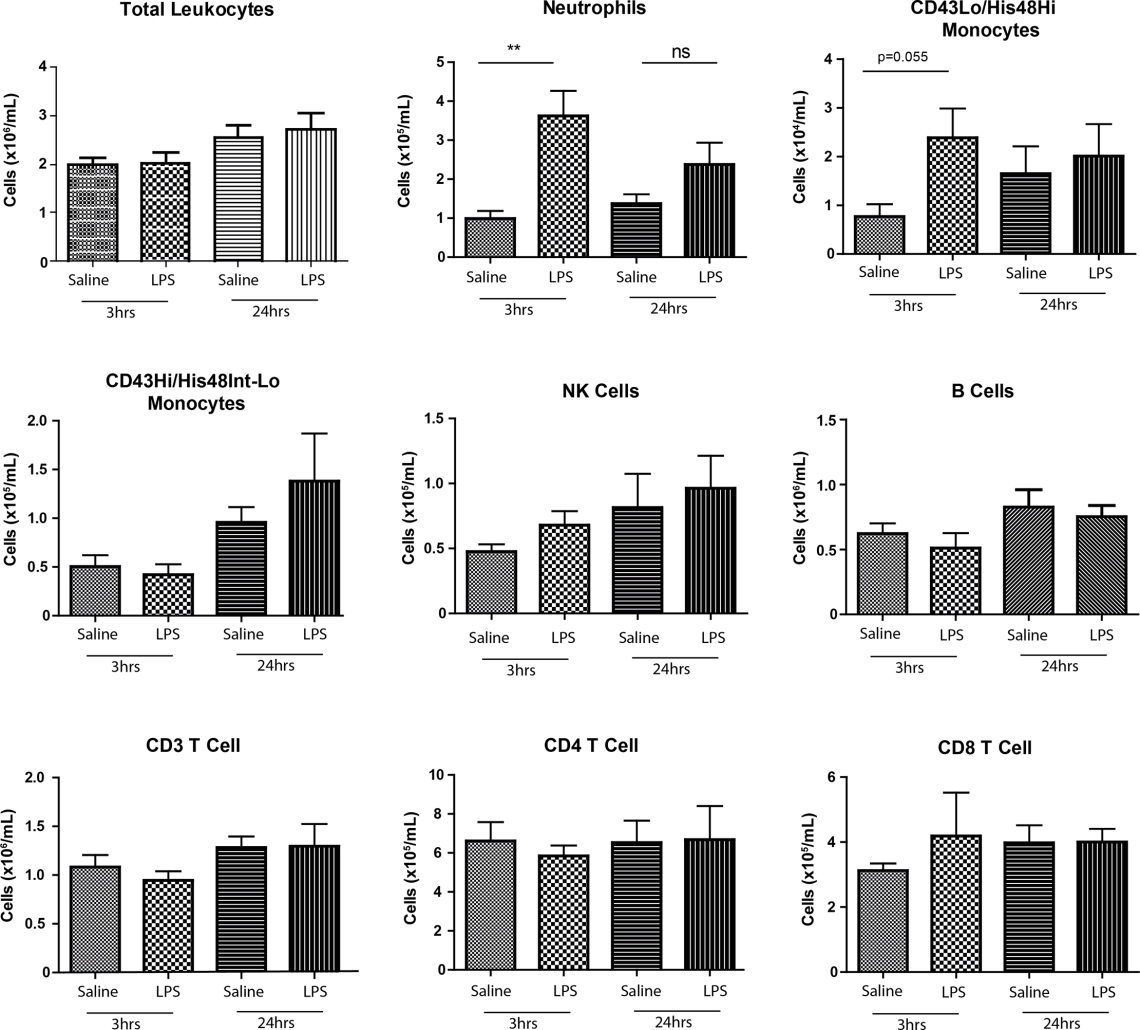
Changes in circulating leukocytes after aerosolised LPS challenge. Rats were exposed to 30mins of aerosolised LPS or saline, at the designated time point blood was collected, processed and analysed. Data are n = 5–6 from 2–3 independent experiments, presented as mean ± SEM where *p<0.05, **p<0.01.

**Fig 4:**
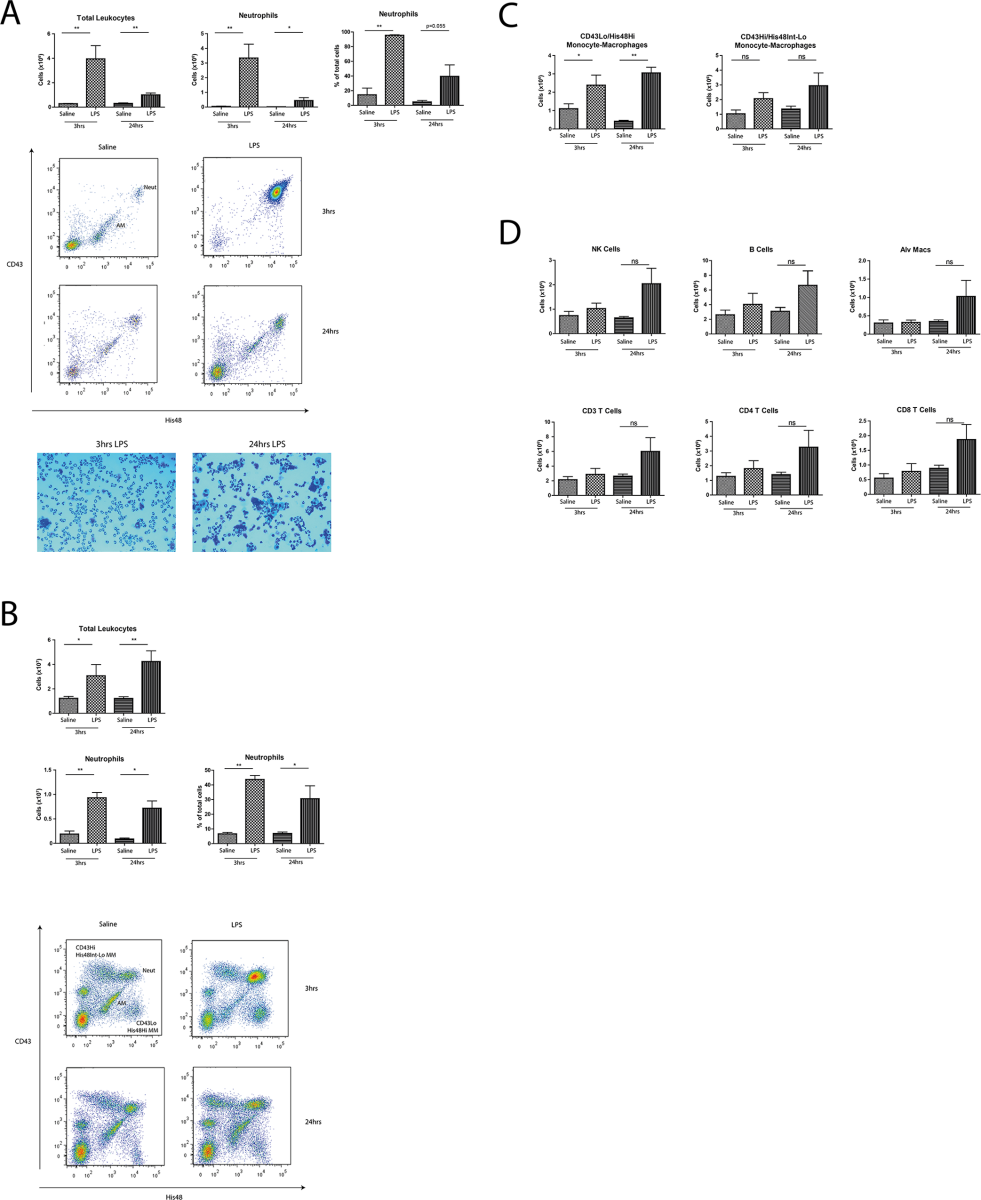
Lung Neutrophils and CD43Lo/His48Hi monocyte-macrophages selectively respond to LPS challenge. BALF (A) and lung (B, C, D) were collected, processed and analysed by FCM at 3 and 24hrs. Data are n = 5–6 from 2–3 independent experiments, presented as mean ± SEM where *p<0.05, **p<0.01.

**Fig 5:**
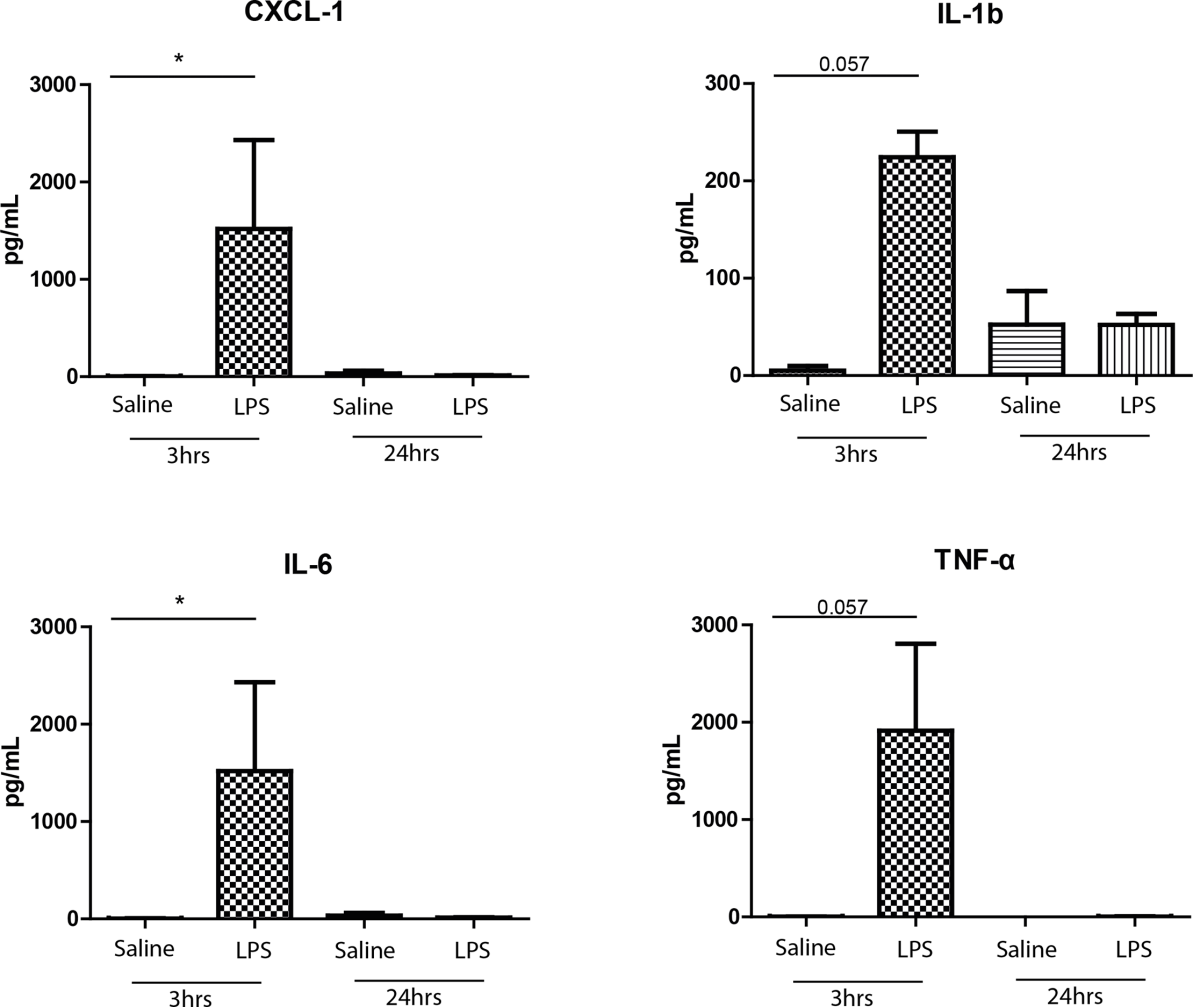
Early pro-inflammatory cytokine release in the BALF following LPS challenge. Increases in pro-inflammatory cytokines were measured in the BAL using an MSD multiplex ELISA according to manufacturer’s instructions. Data are n = 3–4 from 2–3 independent experiments performed in single determinations presented as mean ± SEM where *p<0.05, **p<0.01.

We utilised our 9-colour FCM panel to investigate changes in blood leukocytes after administration of aerosolised LPS to rats (Fig 3). At 3hrs, blood neutrophils were over three-fold higher (Saline: 0.99E5±0.19 LPS:3.6E5±0.64E5 cells/mL, p<0.01) and remained elevated (Saline: 1.37E5±0.56E5 LPS:2.4E5±0.56E5 cells/mL, p>0.05) at 24hrs. We saw an increase in CD43Lo/His48Hi monocytes in the blood at 3hrs (Saline: 0.07E5±0.02E5 cells/mL LPS:0.24E5±0.06E5 cells/mL p = 0.055), but no significant differences amongst CD43Hi/His48Lo-Int monocytes, NK, B or T Cells in the blood at either time point. In the BALF, we saw a significant and selective increase in the total number of neutrophils at 3hrs (Saline: 0.39E5±0.23E5 LPS:33.4E5±9.45E5 cells, p<0.01) and 24hrs (0.14E5±0.19E5 LPS:4.40E5±1.99E5 cells, p<0.05) as confirmed by FCM and cytospins (Fig 4A), together with significant increases in total lung neutrophils at 3hrs (Saline: 18.4E5±6.21E5 LPS:93.0E5±11E5 cells, p<0.01) and 24hrs (Saline: 8.7E5±2.1E5 LPS:71.7E5±14.9E5 cells, p<0.05) (Fig 4B). We observed significantly increased levels of CD43Lo/His48Hi monocyte-macrophages in the lung at 3hrs (Saline:11E5±2.69E5 LPS:23.9E5±5.50E5 cells, p<0.05) and 24hrs (Saline: 4.18E5±0.54E5 LPS:30.5E5±3.07E5 cells, p<0.01) but no significant changes in the CD43Hi/His48Int-Lo subset at either time point (Fig 4C). We saw no other significant differences in NK, B or T Cells or alveolar macrophages (Fig 4D). We then examined pro-inflammatory cytokine release in the BALF; 3hrs after challenge significant increases were seen in CXCL-1 (Saline:4.27±3.05 LPS:1517±914pg/mL, p<0.05) and IL-6 (Saline:4.27±3.05 LPS:1517±914pg/mL, p<0.05) with marked increases in IL-1b (Saline:5.15±4.76 LPS:224±26.2pg/mL, p = 0.057) and TNF-α (Saline:2.74±0.47 LPS:1912±893pg/mL, p = 0.057) (Fig 5).

## Discussion

### Gating strategy

A 9 colour FCM antibody panel has been validated for use in the rat to simultaneously study all major leukocyte populations including neutrophils, B, NK, CD4+ and CD8+ T Cells, AlvMacs and two distinct subsets of monocytes across several tissues. This study is the first to utilise the differential expression of CD43 [10] and His48 on cells to facilitate the delineation of 7 major leukocyte populations across tissues (Fig 1A, 1B and 1C). To our knowledge, no other reported combination of two antibodies permits this in the rat. CD43 is known to play roles in leukocyte tissue emigration and endothelial cell adhesion [19], with reduced expression on neutrophils associated with cellular activation [20]. The functional properties of His48 are less well understood, however it is frequently used as a granulocyte marker [21,22]. We were able to identify Alveolar Macrophages based on their unique pattern of autofluorescence and then confirmed the validity of our gating strategy and panel by sorting and examining cellular morphology (Fig 1D). As shown in Fig 1E our single panel captures the vast majority of CD45+ cells, which in the blood—as expected in the laboratory rat—lymphocytes predominate. This panel could aid rat immunology investigators by serving as a standard for rat leukocyte surface markers in the literature, thereby promoting research consistency; and through economical identification of leukocyte subsets using only 2 or 3 antibodies from this panel, providing greater opportunity to interrogate cell subsets with the remaining PMT channels/antibodies available to investigators.

### Monocyte Characterisation

Monocytes are recruited to and participate in local and systemic inflammatory responses. Two circulating subsets exist in rats and are typically differentiated by CD43 expression alone [10] or with CD172a (SIRP alpha) [23,24]. Despite the growing consensus amongst monocyte investigators in the human and murine literature, this is the first rat study to incorporate monocyte subset delineation into a panel that captures 5 other leukocytes, and demonstrates their distribution across a range of tissues.

Rat monocytes (without sub-set discrimination) have previously been described as ‘SSClow CD4dim’ [4] or ‘CD172a+ TCR-’ [15] by FCM, and by immunohistochemistry using CD68 and CD11b [25] or CD172a alone [26]. CD43Hi monocytes have also previously been shown to be CX3CR1+ CD11c+ and CCR2–, whilst CD43Lo monocytes are CX3CR1lowCD11c– but were CCR2+ [27]. We found each monocyte subset exhibited reciprocal (Figs 1 and 2) expression of CD43 and His48. His48 expression on rat monocytes thus appears analogous to that of Ly6C on murine monocytes [6,11,12].

We characterised the two monocyte populations for common markers (Fig 2B and 2C). CD4 and CD161 are two particular antigens known to decrease and increase in expression respectively during monocyte activation [28], however our data suggest rat monocytes are not CD4 ‘dim’ as described elsewhere [4]. As shown in Fig 2C we found CD43Hi/His48Int-Lo monocytes were CD11c positive and MHC II negative, conversely CD43Lo/His48Hi monocytes were CD11c negative and MHC II positive. Both monocyte subsets were uniformly positive for CD11b, CD172a and CD86 and negative for CD163 as reported elsewhere [23,27,29]. Further, we saw both CD43Hi/His48Int-Lo and CD43Lo/His48Hi monocyte populations distributed across rat tissues (Fig 1C and 1D), particularly in the lung. Due to a lack of firm data regarding these tissue populations, we termed them ‘Monocyte-macrophages’ as reported previously [30–33]. Thus, taken together our data on monocytes aligns with the literature that suggests CD43Lo rat monocytes are analogous to Ly6CHi murine monocytes with the capacity to participate in acute inflammatory responses: a view supported by previous studies in the rat [34,35]. Indeed, CD43Lo monocytes have also been shown in higher numbers at later time points in a model of lung allograft rejection [36] and were isolated directly from the pulmonary vasculature. This highlights the potential for these cells to participate in the inflammatory response from the luminal side of the alveolar-capillary boundary, as well as from within the parenchyma during inflammatory responses.

### LPS Pulmonary Inflammation

To validate our FCM panel and examine monocyte-macrophage specific changes, we used an established aerosolised rat LPS model [37] to investigate cellular inflammation at 3 and 24hrs post-challenge. The leukocyte response to pulmonary inflammation is elicited by the activation of airway tissue pattern recognition receptors. Toll-like receptors (TLR) 2 and 4 are ligated by LPS and evoke signalling that stimulates the release of pro-inflammatory cytokines such as TNF-α and IL-1b from airway tissues, including alveolar macrophages and epithelial cells. This enables leukocyte recruitment by increasing membrane permeability, stimulating chemokine production from endothelial and epithelial cells and promoting upregulation of adhesion molecules on endothelial and immune cells thereby coordinating leukocyte recruitment.

In the rat, this response is well characterised, with neutrophil recruitment seen in airways from as early as 2hrs post-challenge, peaking at 12hrs before beginning to subside by 24hrs, together with significant increases in lung neutrophils [37]. Accordingly, we saw a significant neutrophil specific increase in the blood at 3hrs (Fig 3) with marked increases in CD43Lo/His48Hi monocytes, both of which had diminished by 24hrs. No changes were seen in any other cell types between saline and LPS groups at either time point (Fig 3). As reported previously [37], we saw substantial neutrophil recruitment into the BAL at 3hrs which had subsided by 24hrs (Fig 4A) confirmed both by FCM and cytospins (Fig 4A); together with significant increases in neutrophils in the lung by FCM (Fig 4B).

But interestingly–to our knowledge for the first time in the rat—significant increases at both time points were seen in CD43Lo/His48Hi monocyte-macrophages but not CD43Hi/His48Int-Lo monocyte-macrophages (Fig 4B and 4C). Monocytes are known to respond directly to LPS via the p38 MAPK pathway [38] releasing pro-inflammatory cytokines [37]. Studies show monocytes can infiltrate lung tissue after LPS challenge using both CD11/CD18 and VLA-4 dependant and independent pathways [39], where they may also influence neutrophil lung trafficking [35]. Their depletion prior to LPS exposure can diminish the neutrophil response in a murine model [35] suggesting a key monocyte role in influencing neutrophil-mediated damage in LPS-induced pulmonary inflammation [40]. Taken together, this study presents new insights into the selective role of rat monocyte-macrophages in LPS pulmonary inflammation.

## Study Significance

This study provides a framework for consensus in the rat literature regarding leukocyte identification. It highlights the utility of CD43 and His48 as two versatile markers, including in discriminating neutrophils and CD43Hi and Lo monocytes; and demonstrates the latter’s hitherto unreported selective response to LPS pulmonary inflammation. With other organs, such as the spleen, increasingly recognised as major immunological sites capable of giving rise directly to immune cells such as monocyte-macrophages that participate in distal inflammatory responses [41,42]; this panel holds the potential to advance understanding of splenic and hepatic immune contributions in rat models of disease. Finally, with further research, this panel could be expanded to encapsulate new cells and subsets as they arise.

## Conclusion

In this study we have developed a 9 colour FCM panel to enable the efficient identification and quantification of all major rat leukocyte subsets including classical and non-classical monocytes and monocyte-macrophages. We validated this panel in an LPS model of pulmonary inflammation and demonstrate the selective response not only of neutrophils but also CD43Lo/His48Hi monocyte-macrophages to LPS in a rat pulmonary inflammation model. These monocyte-macrophages were significantly elevated in the lungs at 3 and 24hrs, and may play a role influencing the neutrophil response and onward resolution of inflammation.

## Supporting Information

S1 Supporting Information Minimal data set for Figs 3–5. Data are contained in spreadsheet format according to figures.
(XLSX)
